# lncRNA HOTAIR knockdown suppresses gastric cancer cell biological activities

**DOI:** 10.1002/fsn3.1970

**Published:** 2020-10-30

**Authors:** Pan Chao, Feng Yongheng, Zhou Jin, Zhu Yu, Yang Shiyong, Yang Kunxing, Ma Yong

**Affiliations:** ^1^ Department of General Surgery Nanjing First Hospital Nanjing Medical University Nanjing China

**Keywords:** CCND1, CCND2, gastric cancer, HOTAIR, miRNA‐206

## Abstract

The aim of this study was to characterize the involvement of long noncoding RNA HOTAIR in gastric cancer development. Measurement of HOTAIR and miRNA‐206 expression by in situ hybridization (ISH) and analyzed for the correlation between HOTAIR and miRNA‐206 in gastric cancer tissues. To evaluate the effects of HOTAIR in gastric cancer, MTT assay, flow cytometry, transwell, and wound healing assays were applied. To explain the mechanism behind HOTAIR's involvement, the expression of proteins related to it was also measured by Western blotting. Finally, correlations among related factors were determined by a luciferase target experiment. HOTAIR expression significantly increased, and miRNA‐206 expression significantly decreased in cancer tissues (*p* < .01 and *p* < .001, respectively); HOTAIR knockdown suppressed cell viability, increased cell apoptosis by maintaining cells in the G_1_ phase, and inhibited cell invasion and migration by regulating miRNA‐206 expression (*p* < .01 or *p* < .001). Meanwhile, with HOTAIR knockdown, CCND1 and CCND2 protein expressions were significantly suppressed, whereas miRNA‐206 expression increased (*p* < .01 or *p* < .001). HOTAIR was shown to target miRNA‐206 and miRNA‐206 targeted CCND1 and CCND2. HOTAIR knockdown had antitumor effects by suppressing CCND1 and CCND2 expression by stimulating miRNA‐206 in gastric cancer in vitro study.

## Graphical abstract

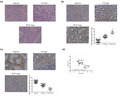

## 1 INTRODUCTION

Long‐chain noncoding RNAs (lncRNAs) are a class of RNAs that are generally defined as nonprotein‐coding transcripts (Yang et al., 2013) larger than 200 nucleotides (nt). In the human genome, more than 15,000 lncRNAs have been found; in contrast, only a few lncRNA features were discovered. LncRNA regulates gene expression at transcriptional and post‐transcriptional levels as well as at epigenetic level, which takes part in the occurrence and development of malignant tumors among others (Liu et al., 2016). Recently, upon revealing the mechanism of prostate cancer, scholars have found an intimate relationship of lncRNA with the incidence and progression, invasion, distant metastasis, and prognosis of prostate cancer (Jiang et al., 2016). It is the time for the public to focus on the role of lncRNA in prostate cancer (Chang et al., 2018; Wan et al., 2018). LncRNA HOX transcript antisense intergenic RNA (HOTAIR) has been conformed to be responsible for the occurrence and development of malignant tumors possibly in the early stage (Cai et al., 2014; Gupta et al., 2010; Pádua Alves et al., 2013). In the beginning, Gupta et al. believed that HOTAIR expression increased in tissues of breast cancer, which was related to the progress of breast cancer (Gupta et al., 2010). According to further research, HOTAIR could guide the repositioning of polycomb repressive complex 2 (PRC2) in genome and thereby cause H3K27 methylation (Gupta et al., 2010; Kim et al., 2013). With respect to gastric cancer, there was a highly expressed HOTAIR in tumor tissues that was found to be strongly correlated with clinical manifestations of those patients such as vascular invasion, lymph node metastasis (LNM), and survival (Endo et al., 2013; Xu et al., 2013). In addition, HOTAIR could combine with miR‐331‐3p in a sponge‐like form and function as the competing endogenous RNA to regulate HER2 expression in gastric cancer cells (Liu et al., 2014). Gupta et al. (Gupta et al., 2010) noticed that high expression of HOTAIR in breast cancer cell lines could change many tumor invasion‐related gene expressions by modifying the complexes with PRC2 chromatin, thus promoting clonal growth and invasion. These scholars also convinced that HOTAIR overexpression in patients with breast cancer was an important predictor of metastasis and poor prognosis (Gupta et al., 2010). In addition, a growing body of literature has reported the universal overexpression of HOTAIR in primary and metastatic tumors of various human cancers including breast cancer (Gupta et al., 2010), hepatocellular carcinoma (Geng et al., 2011), pancreatic cancer (Kim et al., 2013), carcinoma of the lungs (Nakagawa et al., 2013), colorectal cancer (Kogo et al., 2011), and esophageal squamous cell carcinoma (ESCC) (Lv et al., 2013). Moreover, the high expression of HOTAIR was related to the proliferation, invasion, and metastasis of tumor cells as well as the poor prognosis (Wu et al., 2014). Wu et al. (Wu et al., 2014) explored the correlation between HOTAIR overexpression in human cancers and the proliferation, invasion, development, and metastasis of tumor cells as well as the poor prognosis. The purpose of this study is to observe the expression of HOTAIR in gastric tissues and its effect on the biological activity of gastric cancer cells through miRNA.

## 2 MATERIALS AND METHODS

### 2.1 Materials

The materials of study were paraffin‐embedded specimens of 30 gastric cancerous tissues archived after being diagnosed pathologically as gastric cancer and resected by the same surgeon in Nanjing First People's Hospital during the period from 2010 to 2016. Besides, the gastric cancer tissues were collected from surgical margins of the above 30 cases. All patients received no chemotherapy, radiotherapy, or immunotherapy before surgery and were all within the age range of 38 ~ 73 years old. Among them, 20 patients were at stage I‐II and 10 at III‐IV. Other experimental materials included the following: Lipofectamine 2000, TRIzol reagent (Invitrogen, USA), DMEM medium, glutamine, penicillin–streptomycin mixture, fetal bovine serum (FBS) (Gibco, USA), methyl thiazolyl tetrazolium (MTT), dimethylsulfoxide (DMSO) (Sigma‐Aldrich, USA), human HOTAIR‐siRNA (5′‐UUAAGUCUAGGAAUCAGCACGAAGC‐3′), negative control (5′‐UUCUCCGAACGUGUCACGUTT‐3′) (Shanghai GenePharma); in situ hybridization (ISH) kits of HOTAIR and miRNA‐206 (Wuhan Boster), miRNA‐206 mimics, and miRNA‐206 inhibitor (Invitrogen).

### 2.2 ISH assay

After conventional treatment of the tissue microarray in accordance with instructions on the ISH kits, specimens were dropped with prehybridization solution and incubated for 2 hr in the incubator at 42°C. With the absorption of the excess liquid, hybridization solution containing miRNA‐206 or HOTAIR probes was added and then incubated in the incubator at 42°C overnight. The next day, after washing, biotin labeled mouse antidigoxin antibody was added to incubate for 60min in the incubator at 37°C, followed by the addition of SABC and the incubation for another 30 min at 37°C. Following the addition of biotin peroxidase and incubation for 30 min at 37°C, the next steps were DAB coloration, dehydration, transparency, and mounting. Prehybridization solution was used instead of hybridization solution containing miRNA‐206 or HOTAIR probe as negative control. Image Pro Plus digital analysis system was used for image analysis.

### 2.3 HE staining

Following fixation with 10% formaldehyde, the specimens were prepared into paraffin blocks and cut into 4μm slices for hematoxylin–eosin (HE) staining. Pathological changes of prepared section were observed under 200‐fold optical microscope.

### 2.4 Cell culture, grouping, and transfection

Normal human gastric epithelial cell RGM‐1, human gastric cancer SGC‐7901, MKN‐4, BGC‐823, and MGC‐803 cells (Chinese Academy of Sciences Shanghai Institutes for Biological Sciences) were cultured in the RPMI 1640 solution containing 10% FBS and 1% penicillin–streptomycin mixture in a 5% CO_2_ incubator at 37°C. The solution was replaced every other day for routine digestion and passage. Cells in logarithmic growth phase were suitable for further experiment.

SGC‐7901 cells were divided into foour groups: NC, si‐NC, siHOTAIR, and siHOTAIR + miRNA inhibitor groups. SGC‐7901 cells in the NC group were cultured with conventional media; those in the si‐NC group cultured conventionally after transfection of empty vectors; those in the siHOTAIR group were cultured with conventional media after transfection of HOTAIR inhibitor (siHOTAIR); and those in the siHOTAIR + miRNA inhibitor group were cultured with conventional media after transfection of HOTAIR inhibitor (siHOTAIR) and miRNA‐206 inhibitor.

### 2.5 RT‐PCR assay

Total RNA of cells was extracted with TRIzol (Invitrogen, USA) extraction method. lncRNA HOTAIR, miRNA‐206, and U6 primer sequences are shown in Table 1. Relative expression of the target mRNA was indicated with values calculated by 2^‐ΔΔCT^ method. The test was performed 3 times.

**TABLE 1:** The primer sequence

Gene name	Primer Sequence
HOTAIR	F: 5′‐GGGTGGCTCACTCTTCTGGC‐3′
	R: 5′‐TGGCCTTGCCCGGGCTTGTC‐3′
miR−206	F: 5′‐TGGAATGTAAGGAAG‐3′
	R:5′‐GTGCAGGGTCCGAGGT‐3′
U6	F: 5′‐CGCTTCGGCAGCACATATAC‐3′
	R: 5′‐TTCACGAATTTGCGTGTCAT‐3′

### 2.6 MTT Assay of Cell Proliferation

SGC‐7901 cells in the logarithmic growth phase were incubated into the 96‐well culture plate for 24 hr of cell transfection, with 5 replicates in each group. MTT assay was then carried out after transfection for 48 hr. Prior to the end of testing, the MTT solution was added with 10 μl per well. Following continuous culture for 4 hr, the culture solution was discarded and supplemented with DMSO (150 μl/well), followed by shaking for 10 min. The optical density (OD) of each well was measured at wavelength of 490 nm on the Microplate Reader for calculating the rate of cell proliferation.

### 2.7 Apoptosis by flow cytometry

SGC‐7901 cells in the logarithmic growth phase were collected for the preparation of single cell suspension, which were then resuspended in 75% cold ethanol for fixation at 4°C overnight. Cells at the density of 1 × 10^6^ were transferred into the centrifuge tube and resuspended in 200 μl of binding buffer after washing. After that, 5 μl of Annexin V‐FITC and 2.5 μl of PI were supplemented and mixed well, followed by incubation for 15 min at room temperature in dark. With the addition of 400 μl of binding buffer and even mixing, cell apoptosis was measured with flow cytometer (FCM), with the rate of apoptosis expressed as the percentage. The test was performed three times.

### 2.8 Cell cycle by PI staining

After 48 hr of SGC‐7901 cell culture in each group, 100 μl of 75% absolute alcohol was added into each well after the discarding of the supernatant. Following fixation at 4°C overnight and subsequent filtration, cells were rinsed with phosphate‐buffered saline (PBS) twice. All steps were performed in accordance with those described in the instructions on the cell cycle detection kit. As for the specific detection, 100μl of staining solution (RNase 0.2 mg/ml, PI 20 μmol/L) was added into each well and cultured for 1h at 37°C. The scanning was completed by using the Cycle Analysis Template of Acumen Laser Scanning Fluorescence Microplate Cytometry to detect the fluorescence signal excited at wavelength of 488nm. According to the scanning results, the cell cycle was analyzed and the ratio of cell cycle phase was calculated as well. The test was performed three times.

### 2.9 Transwell assay

SGC‐7901 cells of each group in the logarithmic growth phase were inoculated into the 6‐well plate and cultured for 24 hr. Then, cells at the density of 10^5^ (serum‐free) were inoculated into the upper layer of Transwell chambers, with 500 μl of the culture solution containing 10% FBS added into the lower chamber of each group. Cells were then placed an incubator for 24 hr of culture following flat push and even shaking. Following the removal of the chambers and discarding of the culture solution, cells were with PBS 3 times, 3 min each time, and the residual cells were wiped out of the chambers with dry cotton swabs. Fixation was then conducted in 4% paraformaldehyde for 15 min, followed by another washing with PBS for three times. Subsequently, the chambers were placed into the solution with the addition of 0.1% crystal violet in advance and reacted at room temperature for 30 min. After another rinsing with PBS for three times, cell counting and photography were performed under an inverted microscope with random selection of 4 fields of vision.

### 2.10 Wound healing assay

Cells to be measured were inoculated into the 6‐well plate at cell density of about 5 × 10^5^ cells in each well, followed by overnight culture. The cells were completely attached to the bottom surfaces of the wells. The next day, scratches were made with pipette tips against a ruler, followed by cell rinsing with PBS three times and the addition of serum‐free medium for culture. Photography of samples was then performed after 24 hr and 48 hr of culture and sampling to compare the change of cell scratches among groups. Measuring the wound healing rates of different groups.

### 2.11 Western blot assay

Total cell protein was extracted by using protein lysate of RIPA buffer in the experiment group and the control group. The extracted protein was then collected for sodium dodecyl sulfate–polyacrylamide gel electrophoresis (SDS‐PAGE), and the protein on the gel was then transferred onto the polyvinylidene fluoride (PVDF) membrane. CCND1 and CCND2 at the concentration of l: 1,000 were supplemented and incubated in a refrigerator overnight at 4°C. In the next step, rabbit anti‐mouse secondary antibody (1:2000) was supplemented and incubated for 2 hr at 37°C before development with enhanced chemiluminescence (ECL). Differences in protein expression among groups were compared with glyceraldehyde‐3‐phosphate dehydrogenase (GAPDH) as internal reference.

### 2.12 Luciferase target experiment

The wild HOTAIR‐3 'UTR plasmid was constructed, and the mutant vector was constructed by site‐directed mutation technique. Mimics control or miRNA‐206 mimics and HOTAIR‐wt or HOTAIR‐mut vectors were cotransferred into AGS‐7901 cells. Wild‐type and mutant vectors of CCND1 and CCND2 were constructed in the same way. The vector was then cotransfected with miRNA‐206 mimics or analogue controls into AGS‐7901 cells, and luciferase activity was detected by the dual luciferase reporter system (Promega).

### 2.13 Statistical analysis

SPSS19.0 software was utilized for statistical analysis and Spearman for correlation analysis of clinical data. Data of cell experiments were expressed as mean ± standard deviation (*SD*), and the difference among groups was analyzed with one‐way analysis of variance or two‐tailed Student's *t* test. *p* < .05 meant that the difference was statistically significant.

## 3 RESULTS

### 3.1 Clinical data and analysis

**FIGURE 1:**
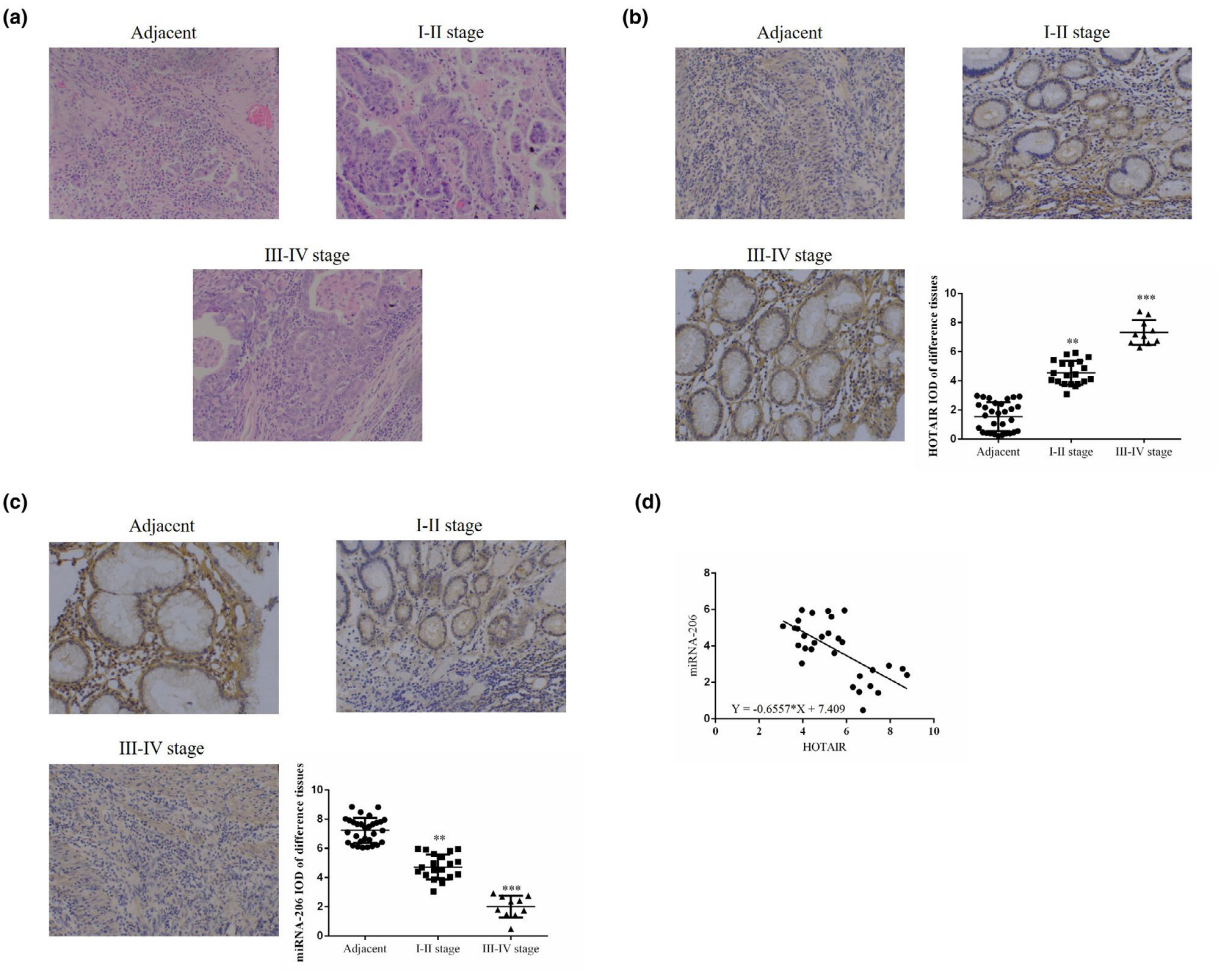
The clinical data and analysis. (a)The pathology of different tissues by H&E staining (200×). Adjacent: adjacent normal tissues; Stage I–II: gastric cancer tissues of Stage I–II; Stage III–IV: gastric cancer tissues of Stage III–IV. (b)HOTAIR expression of different tissues by ISH (200×). IOD: integral optical density. Adjacent: adjacent normal tissues; Stage I–II: gastric cancer tissues of Stage I–II; Stage III–IV: gastric cancer tissues of Stage III–IV. **: *p* < .01, ***: *p* < .001 versus adjacent tissues. (c)miRNA‐206 expression of different tissues by ISH (200×). IOD: integral optical density. Adjacent: adjacent normal tissues; Stage I–II: gastric cancer tissues of Stage I–II; Stage III–IV: gastric cancer tissues of Stage III–IV. **: *p* < .01, ***: *p* < .001 versus. adjacent tissues. (d)The correlation between miRNA‐206 and HOTAIR in gastric cancer tissues

As revealed by H&E staining, cell invasion and migration were increased with increasing cancer stage in gastric cancer tissues, compared with the levels in adjacent normal tissues (Figure 1a). Moreover, as revealed by ISH assay, HOTAIR expression was significantly increased and miRNA‐206 expression was significantly suppressed with increasing stage compared with those of adjacent normal tissues (*p* < .01 or *p* < .001, respectively; Figure 1b,c). Upon analyzing the correlation between HOTAIR and miRNA‐206 in gastric cancer tissues, the results revealed that HOTAIR was negatively correlated with miRNA‐206 (*r* = −.6557, *p* < .0001; Figure 1d).

### 3.2 HOTAIR and miRNA‐206 mRNA expression by RT‐qPCR assay

**FIGURE 2:**
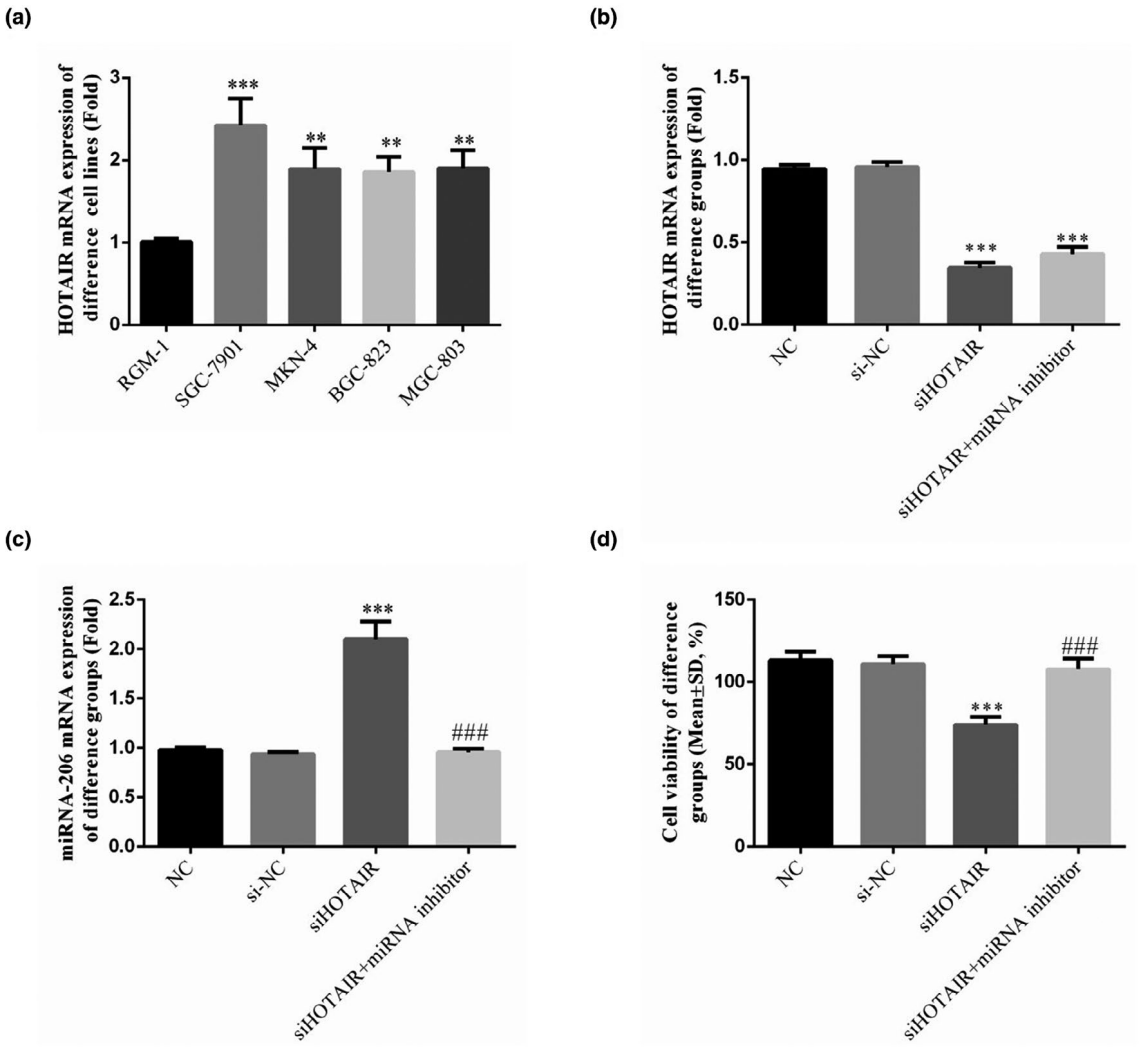
The HOTAIR and miRNA‐206 mRNA expression of different groups and cell lines by RT‐qPCR assay and SGC‐7901 cell viability of different groups by MTT assay. NC: normal control group; si‐NC: SGC 7901 cell transfected with negative control; siHOTAIR: SGC‐7901 cell transfection with siHOTAIR, which inhibits HOTAIR expression; siHOTAIR + miRNA inhibitor: SGC‐7901 cell transfection with siHOTAIR and miRNA‐206 inhibitor. (a) HOTAIR mRNA expression of difference cell lines by RT‐qPCR assay. ***: *p* < .001, compared with NC group; ###: *p *<* *.001; Compared with siHOTAIR group. (b) HOTAIR mRNA of different groups by RT‐qPCR assay. ***: *p *< .001, compared with NC group; ###: *p < *.001; Compared with siHOTAIR group. (c) miRNA‐206 mRNA of different groups by RT‐qPCR assay. ***: *p < *.001, compared with NC group; ###: *p < *.001; Compared with siHOTAIR group. (d) SGC‐7901 cell viability of different groups by MTT assay. ***: *p < *.001, compared with NC group; ###: *p < *.001; Compared with siHOTAIR group

Compared with RGM‐1 cell lines, the HOTAIR mRNA expression of SGC‐7901, MKN‐4, BGC‐823, and MGC‐803 cell lines was significantly up‐regulated by RT‐qPCR assay (*p* < .01 or *p* < .001, respectively, Figure 2a). The HOTAIR mRNA expression of SGC‐7901 cell line was highest in gastric cancer cell lines.

By RT‐qPCR assay, compared with NC group, the HOTAIR mRNA expression of siHOTAIR, and siHOTAIR + miRNA inhibitor groups were significantly down‐regulated (*p* < .001, respectively, Figure 2b). There were no significantly differences between NC and si‐NC in HOTAIR mRNA expression.

The miRNA‐206 mRNA expression of siHOTAIR group was significantly up‐regulated compared with that of NC group (*p* < .001, respectively, Figure 2c); compared with siHOTAIR group, the miRNA‐206 mRNA expression of siHOTAIR + miRNA inhibitor group was significantly depressed compared with that of siHOTAIR group (*p* < .001, respectively, Figure 2c). The relative data were shown in Figure 2b.

### 3.3 HOTAIR had effects on gastric cancer cell viability, as revealed by MTT assay

Upon analyses under an optical microscope, no significant differences in cell morphology were identified among the groups (Figure 2d). The SGC‐7901 cell viability of the siHOTAIR group was significantly reduced compared with that of the NC group (*p* < .001, respectively, Figure 2d). However, the SGC‐7901 cell viability of the siHOTAIR + miRNA inhibitor group that was transfected with siHOTAIR and miRNA‐206 inhibitor was significantly increased compared with that of the siHOTAIR group. There were no significantly differences between NC and si‐NC groups in cell viability. The data on this experiment are shown in Figure 2d.

### 3.4 HOTAIR had effects on SGC‐7901 cell apoptosis, as revealed by flow cytometry

**FIGURE 3:**
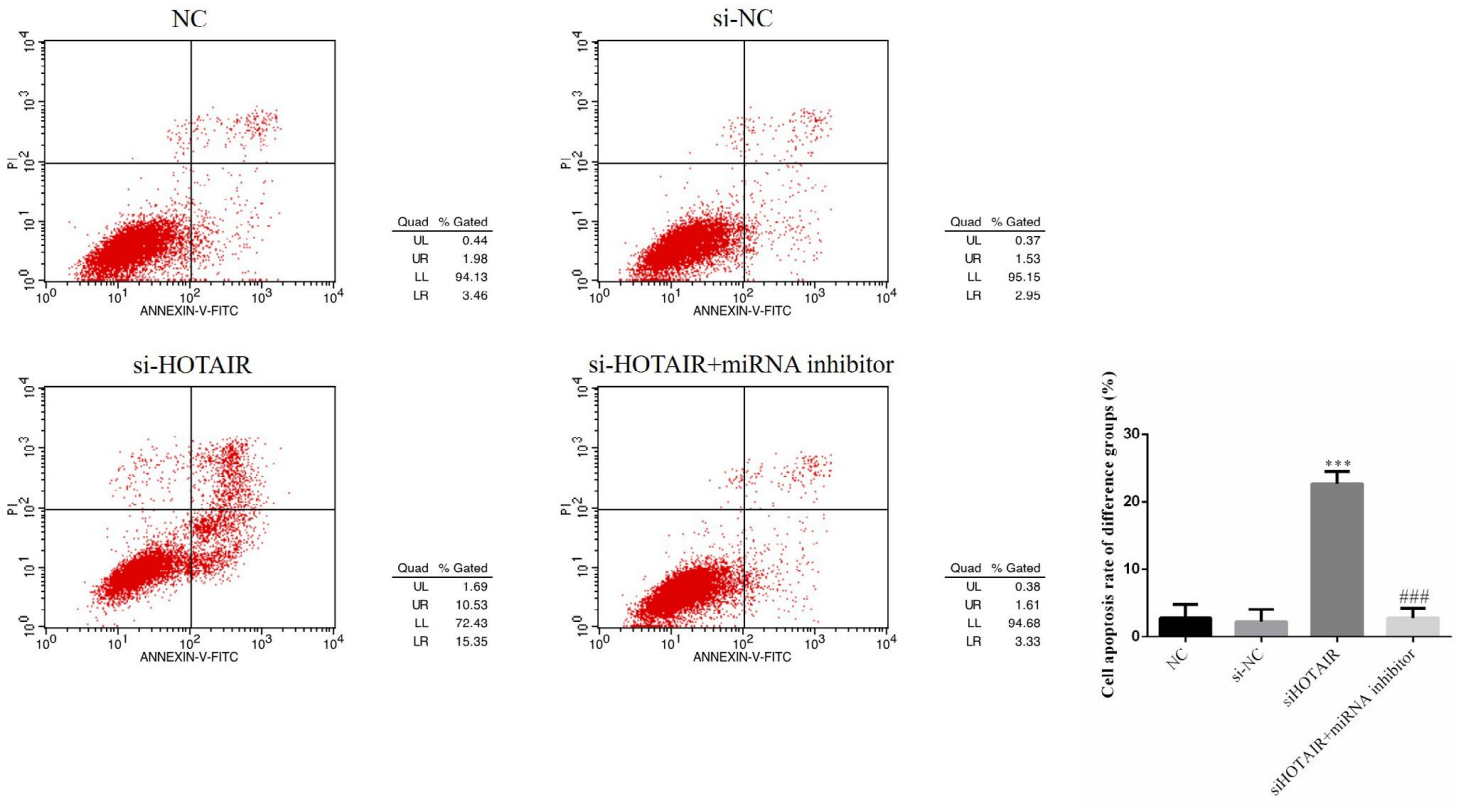
SGC‐7901 cell apoptosis rates of different groups by flow cytometry. NC: normal control group; si‐NC: SGC‐7901 cell transfection with negative control; siHOTAIR: SGC‐7901 cell transfection with siHOTAIR, which inhibits HOTAIR expression; siHOTAIR + miRNA inhibitor: SGC‐7901 cell transfection with siHOTAIR and miRNA‐206 inhibitor. ***: *p *< .001, compared with NC group; ###: *p* < .001; Compared with siHOTAIR group

Compared with that in the NC group, the cell apoptosis rates of the siHOTAIR group were significantly increased (*p* < .001; Figure 3); with miRNA‐206 inhibitor transfection, the cell apoptosis rate of the siHOTAIR + miRNA inhibitor group was significantly suppressed compared with that of the siHOTAIR group (*p* < .001, Figure 3). There were no significantly differences between NC and si‐NC in cell apoptosis. The data on this experiment are shown in Figure 3.

### 3.5 HOTAIR had effects on SGC‐7901 cell cycle, as revealed by flow cytometry

**FIGURE 4:**
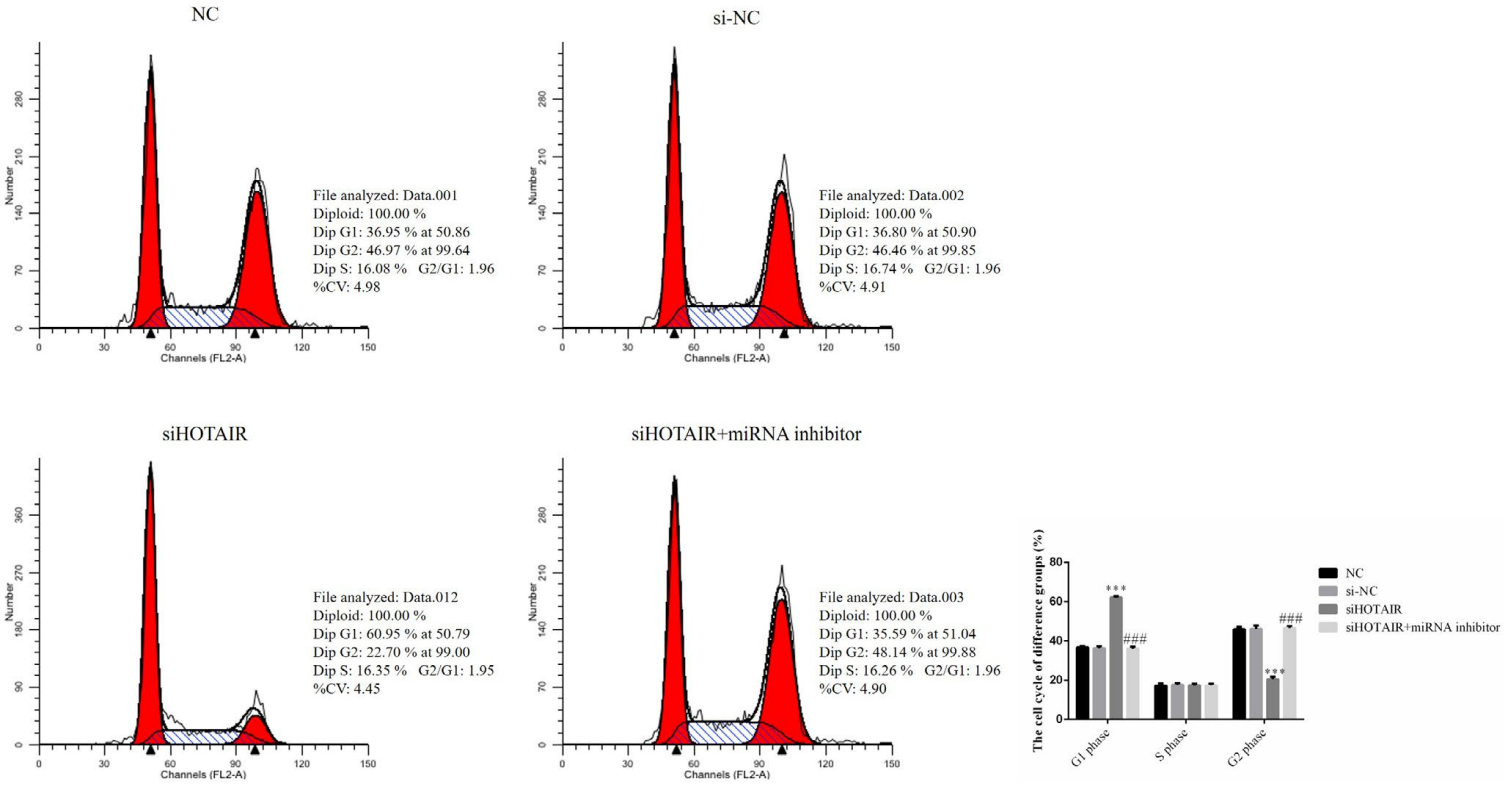
SGC‐7901 cell cycle of different groups by flow cytometry. NC: normal control group; si‐NC: SGC‐7901 cell transfection with negative control; siHOTAIR: SGC‐7901 cell transfection with siHOTAIR, which inhibits HOTAIR expression; siHOTAIR + miRNA inhibitor: SGC‐7901 cell transfection with siHOTAIR and miRNA‐206 inhibitor. ***: *p* < .001, compared with NC group; ###: *p < *.001; Compared with siHOTAIR group

Compared with that in the NC group, the rates of cells in the G_1_ phase in the siHOTAIR group were significantly increased (*p* < .001; Figure 4), without miRNA‐206 inhibitor supplementation; moreover, the rate of cells in the G_1_ phase in the siHOTAIR + miRNA inhibitor group was significantly decreased compared with that in the siHOTAIR group (*p* < .01; Figure 4). Meanwhile, the rates of cells in the G_2_ phase in the siHOTAIR group were significantly decreased (*p* < .001 Figure 4) compared with that in the NC group. Finally, with miRNA‐206 inhibitor transfection, the rate of cells in the G_2_ phase in the siHOTAIR + miRNA inhibitor group was significantly increased compared with that in the siHOTAIR group (*p* < .001; Figure 4). There were no significantly differences between NC and si‐NC groups in cell cycle. The data from this experiment are shown in Figure 4.

### 3.6 HOTAIR stimulates SGC‐7901 cell invasion, as revealed by Transwell assay

**FIGURE 5:**
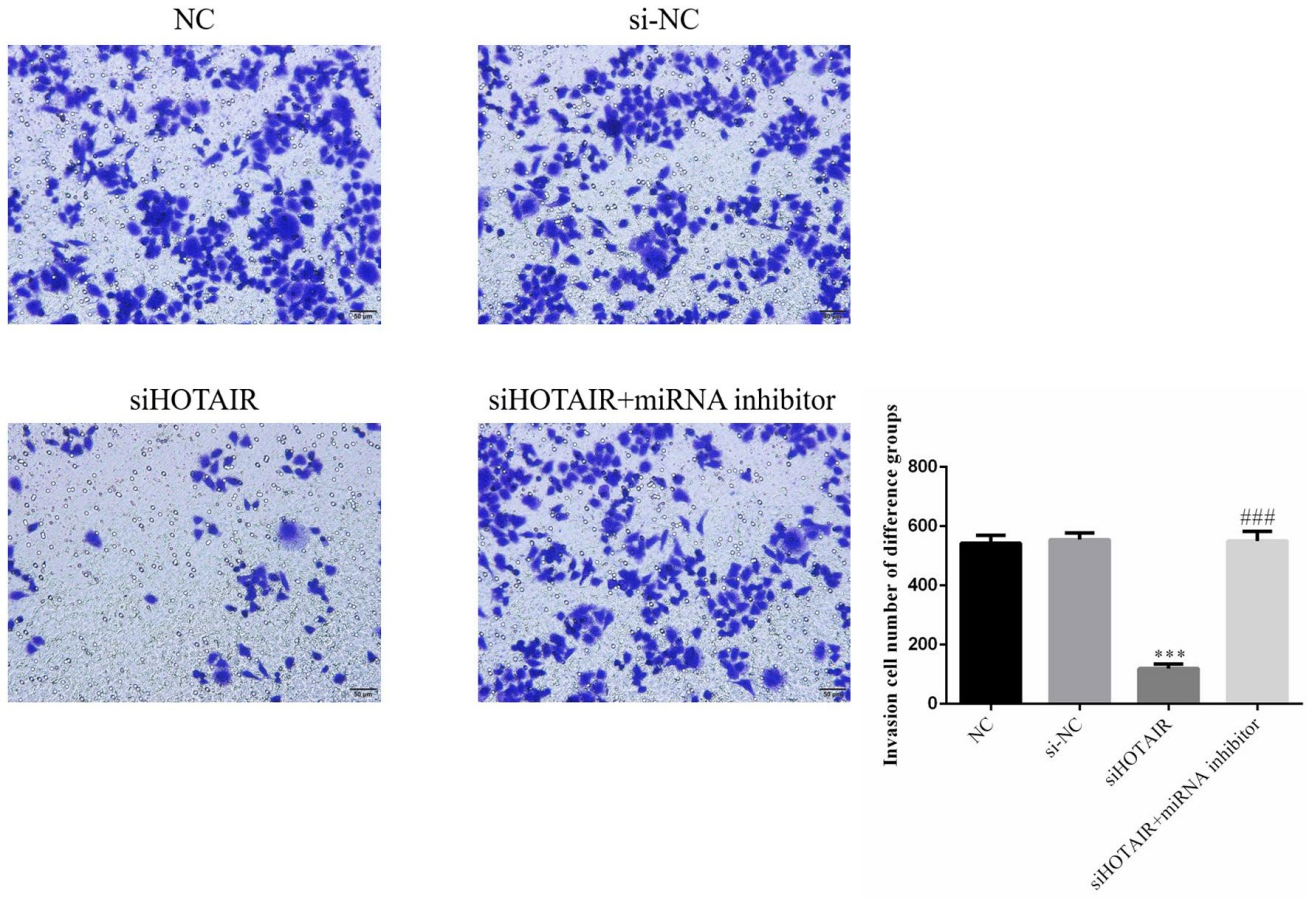
Numbers of invading SGC‐7901 cells in different groups by Transwell assay (100×). NC: normal control group; si‐NC: SGC‐7901 cell transfection with negative control; siHOTAIR: SGC‐7901 cell transfection with siHOTAIR, which inhibits HOTAIR expression; siHOTAIR + miRNA inhibitor: SGC‐7901 cell transfection with siHOTAIR and miRNA‐206 inhibitor. ***: *p *< .001, compared with NC group; ###: *p < *.001; Compared with siHOTAIR group

To explain the effects of HOTAIR on SGC‐7901 cell invasion, the number of invading cells was measured by a Transwell assay. The numbers of invading SGC‐7901 cells in the siHOTAIR group were significantly suppressed compared with that of the si‐NC group (*p* < .001, Figure 5). However, the invasive ability of SGC‐7901 cells was recovered with miRNA inhibitor transfection; compared with that in the siHOTAIR group, the number of invading SGC‐7901 cells in the siHOTAIR + miRNA inhibitor group was significantly increased (*p* < .001; Figure 5). There were no significantly differences among NC and si‐NC groups in invasion cell number. The data from this experiment are shown in Figure 5.

### 3.7 HOTAIR stimulates SGC‐7901 migration, as revealed by wound healing assay

**FIGURE 6:**
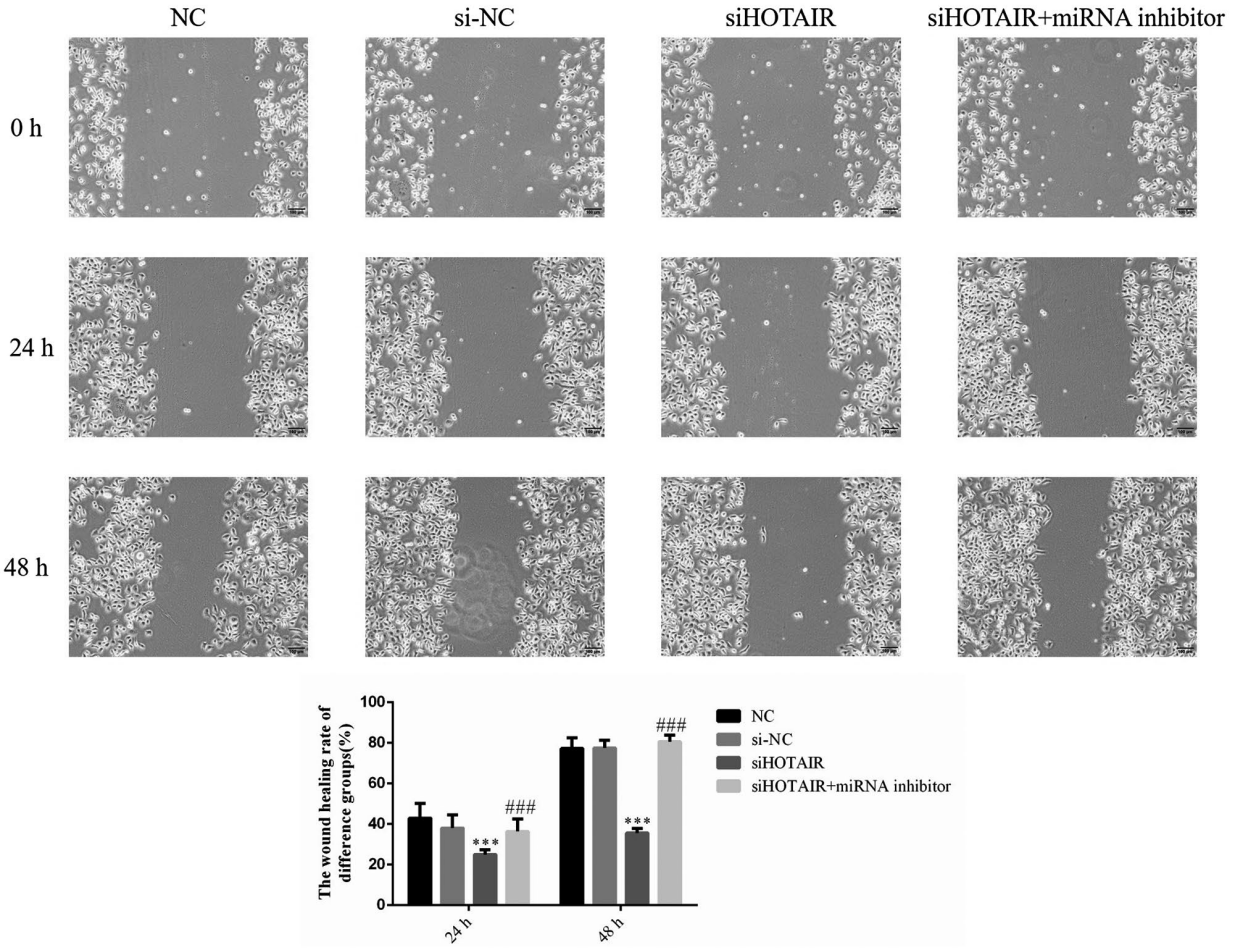
The wound healing rates of different groups at different times (100×). NC: normal control group; si‐NC: SGC‐7901 cell transfection with negative control; siHOTAIR: SGC‐7901 cell transfection with siHOTAIR, which inhibits HOTAIR expression; siHOTAIR + miRNA inhibitor: SGC‐7901 cell transfection with siHOTAIR and miRNA‐206 inhibitor. ***: *p < *.001, compared with NC group; ###: *p < *.001; Compared with siHOTAIR group

To evaluate the effects of HOTAIR on SGC‐7901 cell migration, the wound healing rates in the siHOTAIR group were determined and revealed to be significantly decreased at 24 and 48 hr (*p* < .001, respectively; Figure 6). However, the migration ability of SGC‐7901 cells recovered upon miRNA inhibitor supplementation, compared with that in the siHOTAIR group; moreover, the wound healing rate of the siHOTAIR + miRNA inhibitor group was significantly increased at 24 and 48 hr (*p* < .001, Figure 6). There were no significantly differences between NC and si‐NC groups in cell wound healing rate. The data from this experiment are shown in Figure 6.

### 3.8 HOTAIR affects relative protein expression, as revealed by Western blotting

**FIGURE 7:**
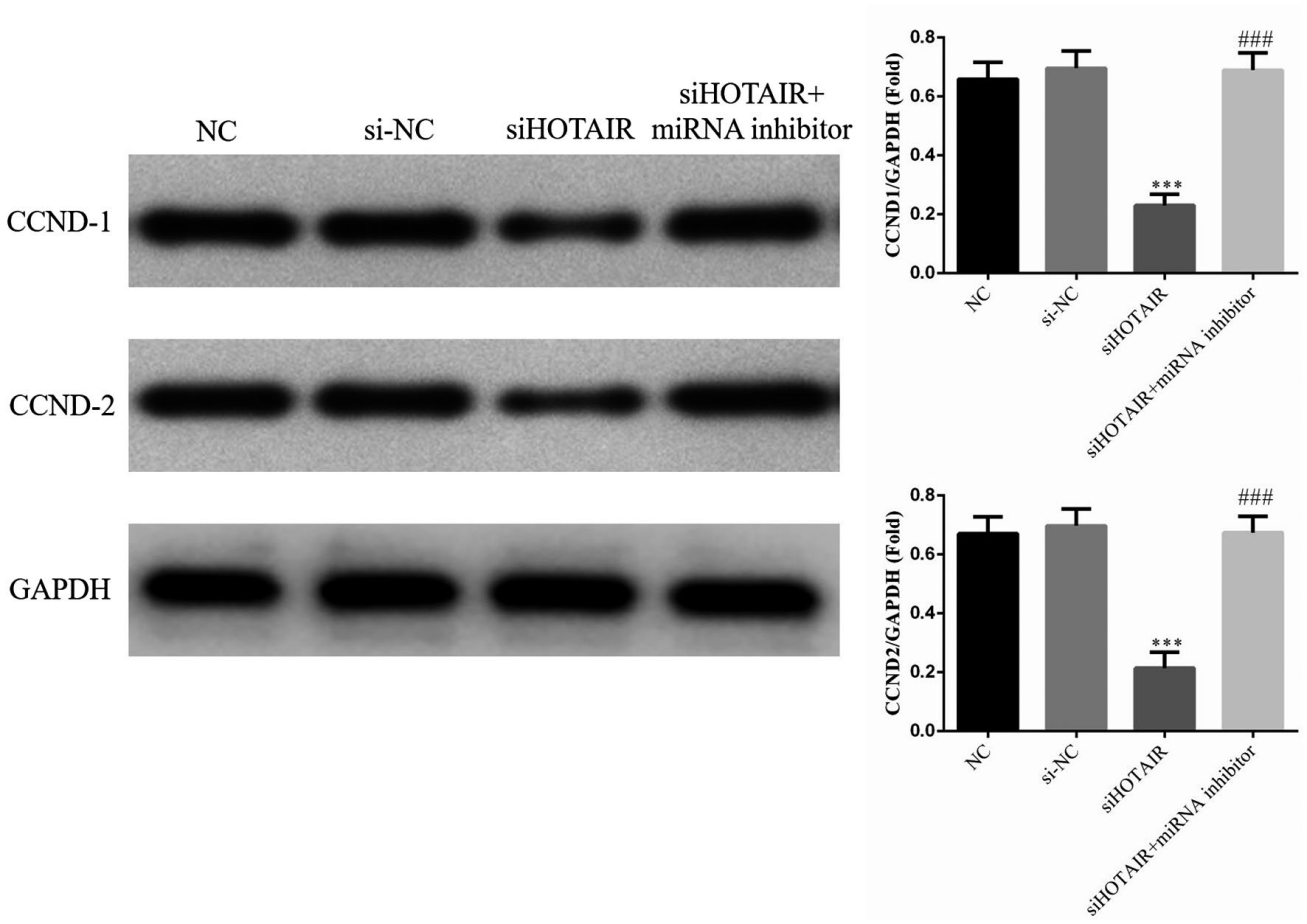
CCND1 and CCND2 protein expression levels of different groups by Western blotting. NC: normal control group; si‐NC: SGC‐7901 cell transfection with negative control; siHOTAIR: SGC‐7901 cell transfection with siHOTAIR, which inhibits HOTAIR expression; siHOTAIR + miRNA inhibitor: SGC‐7901 cell transfection with siHOTAIR and miRNA‐206 inhibitor. ***: *p* < .001, compared with NC group; ###: *p* < .001; Compared with siHOTAIR group

The expression levels of CCND1 and CCND2 proteins in the different groups were measured by Western blotting. Compared with those in the NC group, the CCND1 and CCND2 protein expression levels in the siHOTAIR group were significantly decreased (*p* < .001, respectively; Figure 7). However, the expression levels of CCND1 and CCND2 proteins in the siHOTAIR + miRNA inhibitor group were significantly up‐regulated with miRNA‐206 inhibitor supplementation compared with those in the siHOTAIR group (*p* < .001, respectively, Figure 7). There were no significantly differences between NC and si‐NC groups in CCND1 and CCND2 proteins expression. The data from this experiment are shown in Figure 7.

### 3.9 Testing the correlation among HOTAIR, miRNA‐206 and CCND1/2 by luciferase target experiment

**FIGURE 8:**
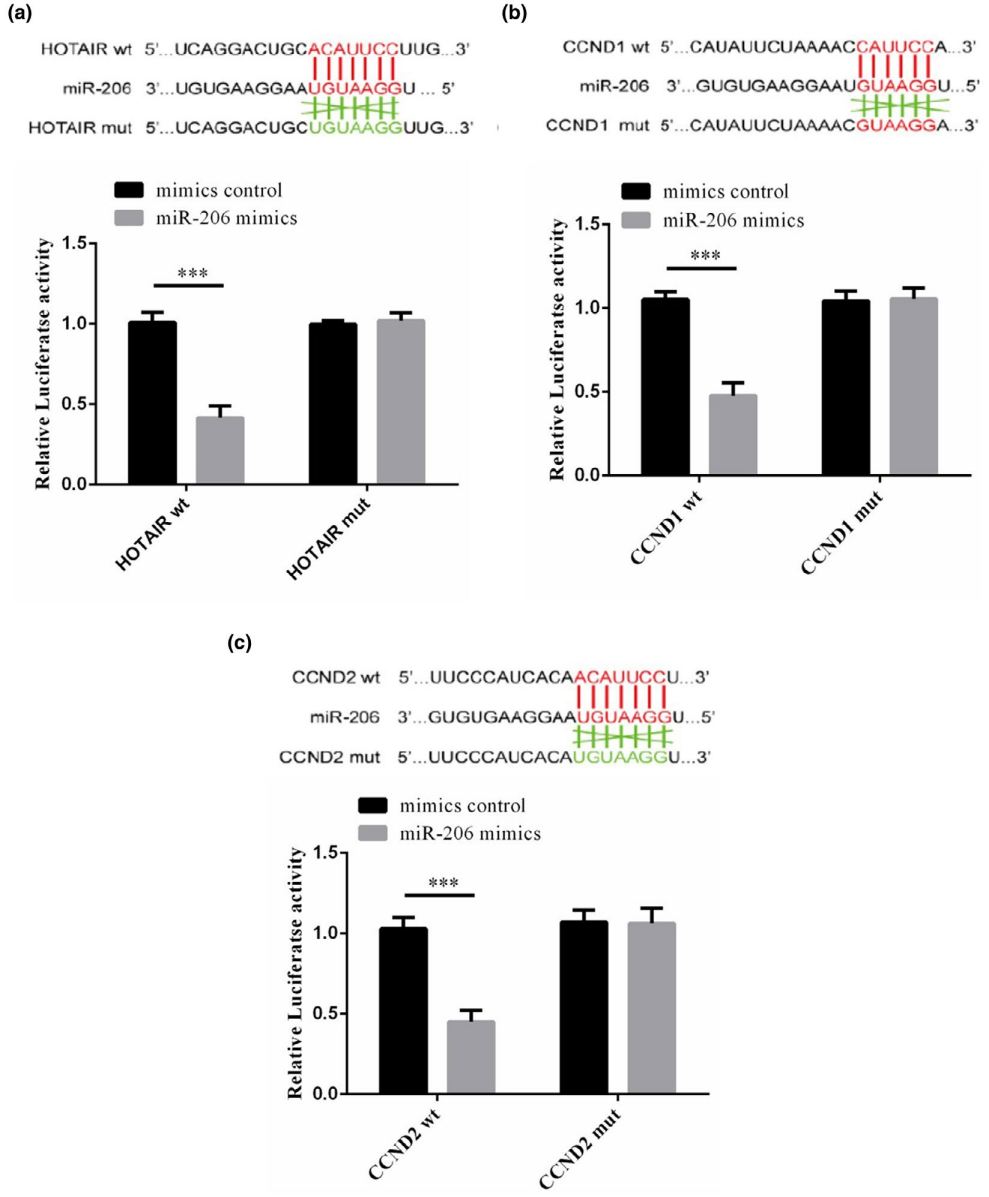
HOTAIR targeted miRNA‐206, which targeted CCND1 and CCND2. (a) The correlation between HOTAIR and miRNA‐206. ***: *p* < .001 versus mimic control. (b) The correlation between miRNA‐206 and CCND1. ***: *p* < .001 versus mimic control. (c) The correlation between miRNA‐206 and CCND2. ***: *p* < .001 versus mimic control

Compared with that in the mimic control, the luciferase activity in the miR‐206 mimic group was significantly suppressed in HOTAIR‐WT (*p* < .001, Figure 8a). Figure 8b shows that the luciferase activity of the miR‐206 mimic group was significantly decreased compared with that of the mimic control group in CCND1 wt (*p* < .001). Figure 8c shows that the luciferase activity of the miR‐206 mimic group was significantly decreased compared with that of the mimic control group in CCND2 wt (*p* < .001).

## 4 DISCUSSION

According to results of the Human Genome Project, protein‐coding genes only account for less than 1% of the total genomic sequence, the remaining being noncoding RNAs (Jiang et al., 2015). LncRNAs take up an important part in noncoding RNAs, which are a kind of nonprotein‐coding RNAs with the length of more than 200nt, generally transcribed in eukaryotic cells. Despite initial recognization as genomic transcriptional noise, lncRNAs have been demonstrated to have message RNA‐like structure and the structure of poly‐A tail and promoters after splicing. However, due to the absence of open reading frames in the sequence, it has no protein‐coding function. Prior evidence has revealed the role of lncRNA in genomic imprinting, chromatin modifications, transcriptional regulation, post‐transcriptional regulation, and many other important regulatory processes (Qiu et al., 2013). LncRNA and protein form complex regulatory network, which exert joint effect on cell proliferation, differentiation, and apoptosis and other vital activities (Fang & Fullwood, 2016). Related research indicated significant difference in lncRNA expression between gastric cancer tissues and normal tissues, suggesting that lncRNA might be a potential marker for early diagnosis of gastric cancer (Gu et al., 2015). Zhang et al. (2017) carried out lncRNA screening and analysis of cancer tissues and adjacent normal tissues in gastric cancer patients and then quantitatively analyzed the related lncRNA of gastric cancer. The research discovered significant increase of plasma lncRNA (TINCR, CCAT2, AOC4P, BANCR, and LINC00857) in gastric cancer cell lines. Moreover, additional studies also supported that lncRNA (H19, LINC00152, UC001lsz) and lncRNA MALATl high expression was related to the increased risk of gastric cancer (Xia et al., 2016; Yang et al., 2016). Recent research also reported the enrichment of lncRNAsAK001058, INHBA‐ASl, MIR4435‐2HG, and CEBPA‐ASl in human gastric cancer tissues and significant increase in the plasma of patients with gastric cancer (Ke et al., 2017).

Related research has proven the high expression of HOTAIR in gastric cancer compared with that in normal tissues (Dong et al., 2019; Jiang et al., 2019). However, it remains unclear with respect to the target miRNA for HOTAIR to act on proteins or signal pathways. In this research, ISH was performed to explore the expression of HOTAIR and miRNA‐206 in gastric carcinoma and adjacent tissues, associated with corresponding correlation analysis. It was found that HOTAIR and miRNA‐206 were negatively correlated. Afterward, cell experiment was conducted to verify whether silencing HOTAIR can inhibit the biological activity of gastric cancer through the downstream proteins medicated by miRNA‐206. Corresponding results demonstrated that HOTAIR silencing could effectively inhibit gastric cancer cell proliferation, invasion, and migration and promote apoptosis by retaining cells in G1 phase. However, miRNA‐206 transfection reversed the antitumor effect of HOTAIR silencing disappeared. Meanwhile, double luciferase reporter gene test verified the targeting relationship between miRNA‐206 and HOTAIR.

CCND1 and CCND2 belong to the D‐type cyclins and are usually considered promoters of human cancer. Up‐regulated CCND1 and CCND2 expression are related to tumor metastasis (Ahlin et al., 2017; Lee et al., 2015; Takano et al., 1999). Meanwhile, the expression of CCND2 has been reported to be increased in chronic B‐cell malignancies and CCND1 elevated in colon cancer, and there is a close relation of CCND1 and CCND2 with clinical results, TNM staging, and metastasis (Delmer et al., 1995; Li et al., 2014). In this research, HOTAIR silencing was effective in inhibiting the expression of CCND1 and CCND2 in gastric cancer cells, which might be related to HOTAIR silence's suppression of the biological activity of gastric cancer. Interestingly, after miRNA‐206 inhibitor's transfection into gastric cancer cells, along with the recovery of the biological activity of SGC‐7901, CCND1 and CCND2 protein expression were enhanced simultaneously. Furthermore, CCND1 and CCND2 were identified as two target proteins of miRNA‐206 with double luciferase reporter gene test. Collectively, our study further clarified that the antitumor effect of HOTAIR silencing in gastric cancer might resulted from the involvement of CCND1 and CCND2 medicated by miRNA‐206.

In conclusion, HOTAIR’s positive regulation of CCND1 and CCND2 in gastric cancer may be realized by reverse regulation of miRNA‐206. Our research supports that targeting HOTAIR‐miRNA‐206‐CCND1/CCND2 signal pathway can be accepted as a potential method for treatment of gastric cancer, providing a new basis for further understanding of the occurrence and development mechanism of gastric cancer.

## CONFLICT OF INTEREST

None.

## ETHICAL APPROVAL

This study was approved by Ethics committee of Nanjing First Hospital.
